# Social resource as a critical and overlooked factor for patient safety in low-resource settings

**DOI:** 10.3389/frhs.2025.1625409

**Published:** 2025-07-03

**Authors:** Hilary Edgcombe, Gatwiri Murithi, Mary Mungai, Stephen Okelo, Sassy Molyneux, Helen Higham, Mike English

**Affiliations:** ^1^ Nuffield Department of Medicine, Health Systems Collaborative, Centre for Global Health Research, Oxford University, Oxford, United Kingdom; ^2^ Safe Surgery and Anesthesia Program, Centre for Public Health and Development, Nairobi, Kenya; ^3^ Anaesthesia Department, Kijabe Hospital, Kijabe, Kenya; ^4^ Department of Surgery and Anaesthesiology, Maseno University, Kisumu, Kenya; ^5^ Nuffield Department of Anaesthetics, University of Oxford, Oxford, United Kingdom

**Keywords:** social support, social resources theory, anaesthetists, low- and middle-income countries, low-resource setting, patient safety, social networks and communities

## Abstract

Clinicians, NGOs, funders and academics (among others) in global health are accustomed to discussion of the “low-resource setting”. Commonly, the resources implicit in this term are physical (equipment, drugs) and infrastructural (electricity, water and sanitation) in nature. Human resources are well recognised as scarce in this context too, and the focus in most “workforce” research is on the number, distribution and/or training of healthcare workers. In this article, we make the case for closer examination of “social resource” as necessary to patient safety and distinct from simple enumeration of available/trained personnel. We use the clinical specialty of anaesthesia as a case study, identifying the different ways in which social resource is necessary to enable safe practice for anaesthesia providers, and the potential challenges to accessing social resource relevant in the low- and middle-income context. Finally, we suggest ways in which social resource for anaesthesia professionals in LMICs might be meaningfully investigated, with a view to improving its priority and access for safe anaesthesia care worldwide.

## 1 Introduction

The phrase “low-resource setting” is a term used to describe challenging contexts for global health, often used as synonymous with or replacement for “low- and middle-income countries” (LMICs). The phrase has advantages—avoiding generalisation by national boundary or national GDP, and allowing for within-country variation—and generally describes limitation in a given context. However, the dominant associations of the phrase “resource”, for most audiences, are likely to be tangible and physical. Imagery (mental and actual) associated with the phrase “low resource setting” in healthcare includes the bare operating room with outdated equipment, tented hospitals in a disaster zone or the al fresco outpatient clinic with a consulting table set up under a tree. Such associations are likely to be at least partly because of the considerable and valuable work done to describe and ameliorate inadequate infrastructural, equipment and pharmaceutical capacity across global health fields (1, 2), but do not tell the whole story. “Human resources” (HR) are also well recognised to be fundamental to healthcare systems and provision, with a tendency for literature and policy to focus most often on the number and distribution of the humans concerned (3). Both these elements are important, but we argue that an additional feature of “human resource”, often overlooked but integral, is the “social resource” available to individuals through their interactions with others.

On the basis of literature review (4, 5), our own experience and pilot fieldwork in Kenya, we propose that social resource is not only a necessary component of healthcare systems but also specifically necessary to providing safe healthcare within those systems, as much if not more so where other resources are limited. In this article we will consider social resource as a concept and its general links to patient safety in healthcare. We will then use anaesthesia as a case study to explore how social resource can be evaluated and investigated in LMICs and/or the “low-resource setting”. An exemplar case illustrating some of the significance of social resource in and around an emergency situation is shown in Table 1.

**Table 1:** Different social resources, the same patient.

	A district hospital in a high-income country	A district hospital in a low-income country
Presentation	Mrs. M presents for urgent Caesarean section at term. She has had two previous sections elsewhere, and the last one was described as “stressful” according to her husband. She has just had a scan which confirms placenta praevia and possible accreta. She has mild anaemia. Her airway looks potentially difficult to intubate.	
Pre-operative management	Dr. X, the anaesthesia resident, assesses Mrs. M and identifies a high risk of bleeding, which their consultant, Dr. Y, confirms. They discuss the situation with the obstetric team and prepare for major haemorrhage. Dr. Y also consults an experienced colleague, Dr. Z, who suggests pre-operative precautions and agrees to be present during surgery. Roles for managing significant haemorrhage are allocated in advance.	Miss X, the clinical officer anaesthetist, assesses Mrs. M and identifies a high risk of bleeding. She consults with Mr. Y, the only other anaesthesia provider at the hospital who is on leave and travelling, but reachable for advice. Miss X contacts the blood bank, which has two units of type O blood available. She attempts to call the physician anaesthesiologist at the nearest regional referral hospital but receives no answer.
Progress	Mrs. M is prepared for surgery under spinal anaesthetic. All proceeds uneventfully until delivery of the baby, who appears white and floppy. The surgical suction fills with blood and the obstetrician tells the team that there is “a lot” of bleeding. Mrs. M becomes rapidly unresponsive with intermittent airway obstruction, BP of 50/30 and HR of 160.^a^	
	The three anaesthetists adopt their planned roles: Dr. Y gives general anaesthesia drugs and intubates Mrs. M. It is not easy so Dr. Z immediately assists, securing the airway. Meanwhile Dr. X is responsible for giving blood products and fluids as fast as possible. Dr Z spots that medications to contract the uterus have not yet been given: this omission could be contributing to the ongoing bleeding. She alerts the team and gives them. Dr Y sets up infusions to support blood pressure, gives drugs to improve clotting function, sites additional intravascular access and takes blood samples to evaluate current status. Dr Z maintains an overview of events, assisting where necessary, communicating with the surgical team, the paediatricians looking after the newborn, and the laboratories providing blood products, and checking to make sure nothing else is missed during the chaotic scene.	Miss X tries to decide what to do first: the obstetrician hands her the baby for resuscitation while scrambling to try and control what looks like torrential bleeding. Mrs. M's oxygen levels drop, the fluid bag is empty, and the monitor can't read her blood pressure. Miss X calls Mr Y, who is too far away, and the lab, who do not answer. With everyone else busy, she urgently calls for a midwife to help with the baby. She inserts another IV line and administers more fluids, wishing she had blood available. She does not think she will be able to intubate Mrs M, so does not dare to risk a general anaesthetic, but fears she may be aspirating. She worries about Mrs. M's deteriorating condition and her weak pulse, contemplating the possibility of another mortality under her care.
Progress	With some difficulty, the obstetricians control the bleeding after about 20 min. They close the uterus and abdomen. Mrs. X has received a lot of fluid and appears puffy around the face, pale and has cold arms and legs. She remains hypotensive despite all efforts.	
	Drs. X, Y, and Z discuss Mrs. M's case and agree that she requires post-operative critical care. They contact the regional centre, who confirm they can accommodate Mrs. M. She is transferred intubated, accompanied with Dr. Y while Dr. Z provides cover for theatres during Dr. Y's absence from the hospital. When Dr Y returns, they conduct a debrief. Dr. X subsequently presents the case at the local morbidity and mortality meeting, leading to improvement in protocols for “suspected accreta” delivery and measures to prevent drug omission errors. Dr. Z conducts informal follow-ups with both Drs. X and Y to monitor their emotional well-being and address any concerns regarding the events. All three anesthesiologists reflect on the teamwork and stress involved in the situation, expressing gratitude upon learning that Mrs. X made a full recovery and returned home.	Miss X and the surgeon concur that Mrs. M requires critical care support. Miss X contacts the referral hospital, but after multiple transfers, it is confirmed that there are no available beds. She attempts to monitor Mrs. M in the theatre; however, due to the urgency of three other patients requiring immediate sections, Mrs. M is transferred to the ward. Later in the evening, while still in theatre, Miss X is informed that Mrs. M's condition has further deteriorated. Miss X is unable to assess Mrs. M immediately as she is administering anaesthesia. Shortly after, the ward nurse reports that Mrs. M has experienced another haemorrhage and has passed away. The anaesthesiologist returns Miss X's call, having previously been busy with another case. Miss X asks herself, again, what she did wrong, but there is no one to review the case with her as the surgeon and family criticize her care. Exhausted and overwhelmed, she continues her singlehanded on call duties without time for reflection, wondering how much longer she can keep going. She thinks again about moving on.

^a^ BP, blood pressure; HR, heart rate.

## 2 Social resource is intertwined with patient safety

### 2.1 “Social resource” as a concept

The term “social resource” has a multiplicity of definitions which are beyond the scope of this article. Instead we highlight here two key approaches to understanding the concept. Firstly, the term may refer to the actual resource(s) exchanged between individuals, which have been categorised in various ways, for example as love/affection, status, information, services, goods or money (6). “Functional” theories of social resource which examine the purposes and results of resource exchange often assume or incorporate this approach. Secondly, “social resource” can describe the social contexts and networks within which exchange occurs or is facilitated, constituting a more “structural” approach. Social resource theories vary in their emphasis on one or both approaches, and include, among many others, social exchange theories (SET) which focus on the give-and-take of resources between or among people (7), job-demands-resources (JD-R) theory which addresses the influence of job demands and resources on employee wellbeing and performance (8), conservation of resources theory (COR) which stresses the efforts made by individuals to maintain valuable resources (9) and network theory, which originates from both sociologic and mathematical disciplines (10) to explain how networks of relationships between individuals influence outcomes. Alongside these (and many other) theoretical approaches lies a body of linked work using the term “social support” which again has been variably defined including both structural and functional aspects (11, 12). It is our view that, their considerable heterogeneity notwithstanding, social resource perspectives provide valuable insights into safety within the healthcare context.

### 2.2 Social resource, health care and patient safety

Much work examining social resource in a healthcare context has focused on the social resource of patients. This has frequently been evaluated with a view to understanding how patients’ social context influences their healthcare outcomes in fields such as breast cancer (13), heart disease (14) and diabetes (15), and often concludes that social resource is of positive benefit to patients.

However, the social resource context of healthcare workers (HCWs) also affects patient outcome, through multiple mechanisms. Individuals' wellbeing, job satisfaction and retention are safety mediators which are affected by social resource availability (16–18). In the clinical context, “speaking up” and “safety voice” behaviours are enhanced by peer support availability (19). Engagement with clinical improvements (18) and patient safety climate (20) are improved by stronger social capital, and cohesive, collaborative professional networks enhance quality and safety of care (21). Other approaches in the patient safety field explore the quality of social interaction between colleagues [e.g., the role of civility/rudeness (22), or the Appreciative Inquiry approach (23)].

Organisational culture and climate have also been increasingly attended to in health systems for their likely relevance to patient safety outcomes (24). Constructs differ but tools used to evaluate organisational culture frequently include elements linked to or dependent on social resource among colleagues such as support, teamwork and collaboration (25, 26). “Organisational software” elements such as the relational environment of workers are necessary to health systems' function (5), ability to implement effective intervention (27) and everyday resilience (28): thus they are likely to have significant impact on patient outcome.

## 3 Anaesthesia as a case study

Anaesthesia as a clinical specialty provides a useful case study of the various ways in which social resource availability at work can influence patient safety within an acute healthcare setting. Anaesthesia providers (APs) work within a surgical team in theatres, taking responsibility for the safe delivery of anaesthesia to patients requiring surgery. This starts with decision-making pre-operatively with the patient, surgeon and other parties oriented toward risk evaluation and shared understanding of the treatment options available. In theatre, the anaesthesia provider works on a short feedback loop, continuously monitoring and responding to events over narrow timeframes. Some patients are critically ill and complications can be anticipated. In other cases complications may occur unexpectedly with limited time for an appropriate action by the AP. Because of the high acuity and reliability requirements for safe anaesthesia, historically anaesthetists have embraced patient safety learning and recognised the key role of human factors and interactions in mitigating the effects of anaesthesia and its complications. In high-income countries, the anaesthesia department is generally one of the largest in a hospital; therefore although communication between providers is sometimes examined for how it could be best used to enhance safety, the availability of those providers for communication is generally assumed.

### 3.1 Social resource and safety in anaesthesia

It is clear that the availability of social resource to anaesthesia providers from within their own specialty is potentially relevant to patient safety in several ways. Most acutely, another provider may be called upon to offer immediate assistance with the management of a critically ill patient, either for their specific expertise, simply as another skilled “pair of hands” or as a “fresh pair of eyes” able to reassess a situation and evaluate what needs to be done. In a less urgent clinical context, patient safety may be enhanced where an AP can seek advice (often in structured preoperative assessment clinics), “sense-check” a plan, or ask for additional presence prior to undertaking a challenging case. Outside the operating theatre APs communicate with one another for teaching, training, mentoring and professional development activity which all potentially enhances patient safety. Structured morbidity and mortality reviews with peers, or informal feedback and reflective conversations, enable providers together to identify system-based learning from safety incidents (which may include errors), and improve safety for subsequent patients. APs struggling after adverse events such as intraoperative mortality may be safer and more likely to continue in their jobs if supported by colleagues (29), as well as less likely to adopt harmful coping strategies such as substance abuse (30). While APs (as any other healthcare provider) will also seek social resource from outside their own profession, whether from other HCWs in the team or from wider friends and family, the areas described above are those where social resource from within the same profession confers distinct benefit.

### 3.2 Social resource availability in anaesthesia in LMICs

Thus social resource mediated by interaction between anaesthesia providers has substantial relevance to patient safety. Unfortunately, the availability of colleagues so commonly taken for granted in high-resource contexts cannot be similarly assumed in low-resource settings. There are vastly fewer anaesthesia providers per head of population in many LMICs, meaning that both the number and distribution of anaesthesia providers in many countries is insufficient to staff all hospitals which aim to provide surgical care (31, 32). In rural and remote areas there may be only one or two anaesthesia providers working at a given hospital, managing a huge burden of disease. The pressures of daily work may crowd out thoughts of engaging social resource, even if it were available (33). In urban areas, multisectoral working is common (34): providers may work at a government institution but also undertake cases *ad hoc* at private hospitals where they may be the only AP on site. Formal systems are not always in place to support inter-AP communication through “on call” structures, especially in smaller privately run facilities.

In many LMICs, the AP workforce is also multi-cadre, with mid-level providers such as clinical officers, or nurse anaesthetists, providing the majority of care (31). Discourses around “task-sharing” (a concept which implies communication between such providers and supportive physician anaesthesiologists) (35) have not generally examined the potential limitations on and mechanisms of such communication (35). Studies evaluating patient safety associated with anaesthesia provision by different cadre groups have generally focused on comparisons between physician and non-physician anaesthesia providers' outcomes, rather than the interaction between providers (36). In rural contexts, resources and referral options may be especially limited, making consultation opportunities crucial to effective clinical management (37). We note the value placed on presence of another anaesthesia provider in the rural context which was identified in recent discrete choice experiments conducted in Uganda with physician anesthesiologists (34).

The relative scarcity of anaesthesia providers in LMICs compared with HICs, and in rural and remote areas compared with urban centres, makes maximising their effective access to social resource potentially even more valuable in mediating patient safety and other desirable outcomes.

### 3.3 Investigating social resource for anaesthesia providers in LMICs

The investigation of social resource is a distinct approach to workforce planning which builds on and develops our understanding of how workforce capacity can be improved. Most studies of the anaesthesia workforce in any context, but particularly in LMICs, relate to the fundamental question of quantification: how many providers are available, and needed, for adequate surgical care provision (38). A small body of literature also relates to the expansion of training programs, for example, comparing programs within a region (39) or describing specific initiatives (40–42). Very little work to date has described the processes by which anaesthesia providers work together and build capacity (the mechanics of task-sharing), which must be a key element of optimising workforce effectiveness. The evaluation of access to and impacts of social resource provides a framework to move toward this end.

There are several different ways in which the potential scope, impact and means of social resource for anaesthesia providers could be investigated, which have distinct assumptions and audiences, dependent on the methodological approach taken. The scope of social resource for anaesthesia providers refers to the different situations in which social resource might be accessed or needed within their working context. We propose that a clearer understanding of the different situations in which social resource is used or wanted by anaesthesia providers (for example, the ability to get help in a clinical emergency vs. the ability to seek emotional support after a distressing case) could allow the conceptualisation of distinct “social resource pathways”. Such pathways might differ in features such as time-criticality, or who support is best provided by, resulting in different challenges and solutions to improving social resource access. Pathways relevant to providers in low-resource settings or LMICs should be defined using data grounded in the experience of such providers for maximum contextual relevance and potential for positive impact.

**Figure 1:**
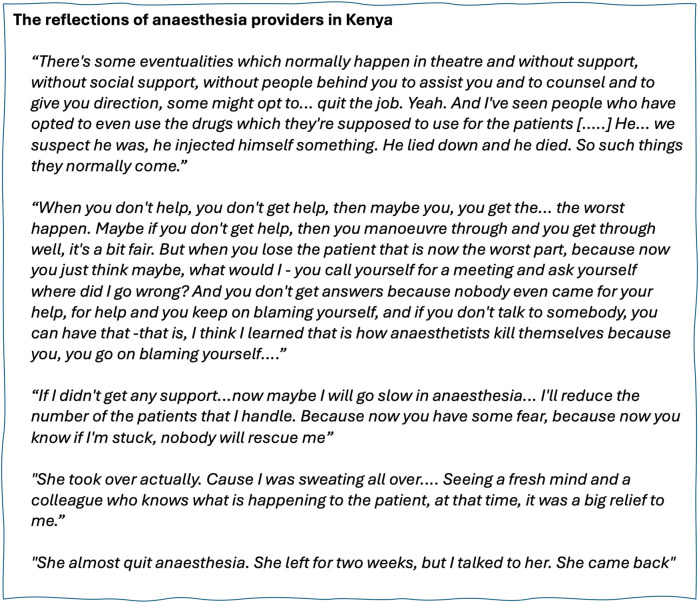
The impacts of social resource. Quotations from anaesthesia providers retrieved as part of pilot fieldwork.

The possible impacts of social resource accessibility for APs relate to direct patient outcomes, indirect patient outcomes and provider wellbeing, and all have relevance to patient safety. Provider wellbeing has been an increasing focus in recent anaesthesia literature, often using “burnout” as the key marker of wellbeing: the majority of literature has aimed to identify its prevalence and association with variables such as gender or level of experience of the anaesthetist, with variable methods and conflicting results (43). The wider impacts of social resource access for anaesthesia providers on their patients, colleagues, teams and communities are unknown. Our group, working between the UK and Kenya, has started to investigate how anaesthesia providers in Kenya, from all cadres, view their social resource and access to it. We conducted preliminary fieldwork with twelve anaesthesia providers from all cadres represented in Kenya [clinical officer anaesthetists (CO-As), Kenyan registered nurse anaesthetists (KRNAs) and physician anesthesiologists (PAs)] to explore whether and how support at work from colleagues is important in their practice using semistructured in-depth interviews. This data identified the high value placed on support from colleagues for anaesthesia providers in both district and tertiary hospital contexts, with clear relevance to patient safety in time-critical emergencies, in learning and seeking advice pre-operatively, and in mediating the wellbeing of providers attempting under considerable constraints to provide safe anaesthesia care (Figure 1). The exemplar scenario found in Table 1 draws from the preliminary data as well as the personal experience of the authors to illustrate this value.

Finally, the social resource available to APs in LMICs is likely to be variable in quantity and quality, and could depend on several different factors, from the individual (e.g., level of experience, how long they have worked in the same place), work-related factors (e.g., the size of the hospital and anaesthesia department, the cadres present providing anaesthesia, departmental norms and hierarchies related to reaching out for help or advice) and institutional or regional factors (e.g., the existence of systems for seeking and providing social resource such as clinical advice for subspecialist care). We suggest that a qualitative approach to understanding the ways in which different social resource processes operate in different contexts, could provide insights into “what works” and “what could work” to optimise the availability and effectiveness of social resource in LMIC contexts.

## 4 Conclusion

We propose that social resource is a key workforce concept relevant to understanding and developing the scope and safety of anaesthesia provision in LMICs, alongside and building on the existing approaches to quantifying the workforce and developing training capacity. Approaches which investigate the scope and describe the impacts of social resource are likely to be necessary to engage stakeholders and decision-makers. Understanding the processes and key determinants of social resource access for anaesthesia providers will provide the basis to identify feasible interventional approaches and foci to improve.

## Data Availability

The datasets presented in this article are not readily available because of the nature and sensitivity of the qualitative data presented. Requests to access the datasets should be directed to anae0170@ox.ac.uk.
